# Rare Incidental Finding of Multiple Pseudoaneurysms After Imaging-Guided Kidney Biopsy in a Patient With Lupus Nephritis: A Case Report and Literature Review

**DOI:** 10.7759/cureus.72659

**Published:** 2024-10-29

**Authors:** Katherine E Guardado, Monte Harvill, Aniruddh Mannari, Christopher Kelly

**Affiliations:** 1 College of Osteopathic Medicine, Michigan State University, Lansing, USA; 2 Diagnostic and Interventional Radiology, Detroit Medical Center, Detroit, USA; 3 Radiology, Detroit Medical Center, Detroit, USA

**Keywords:** embolization, interventional radiology, kidney, renal pseudoaneurysm, vasculitis

## Abstract

Renal pseudoaneurysms are serious and rare vascular lesions. They can be seen in vasculitis or, more commonly, after renal biopsy, percutaneous renal surgery, penetrating trauma, and less frequently due to blunt renal trauma. We present the case of a 28-year-old woman with lupus nephritis accompanied by the incidental finding of renal pseudoaneurysms in both kidneys after an elective percutaneous renal biopsy of the left kidney. An abdominal CT scan showed a subcapsular hematoma and likely intraparenchymal lower pole hematoma. Two days after the procedure, angiography showed multiple pseudoaneurysms in the left kidney as well as a pseudoaneurysm in the upper pole of the right kidney.

## Introduction

Percutaneous renal biopsy (PRB) is an essential procedure for diagnosing and prognosis of renal disease, guiding management, and monitoring renal transplant function [1]. Iversen and Brun introduced this procedure in 1951 [1]. Ultrasound and CT guidance and automatic core biopsy systems have enhanced tissue adequacy and safety of the procedure [2]. The rate of severe complications leading to patient demise has decreased from 0.12% to 0.02% [2]. The primary indications for native PRB include evaluation of proteinuria, microscopic hematuria, renal manifestations of systemic disease, and unexplained renal failure [2]. 

Bleeding complications are common due to the vascularity of the kidney and the inability to compress the biopsy location, given its deep retroperitoneal location [2,3]. In a study of 617 patients who underwent percutaneous ultrasound-guided biopsy, post-biopsy bleeding was common, occurring in 12.8% of patients. However, most cases were of no clinical significance. Only 1.6% required a blood transfusion, and 0.3% required invasive management. Detection of these events was immediate in most patients (92.4%) [1]. In addition, it has been reported that patients with a vasculitis-related disease have a high probability of significant bleeding after percutaneous renal biopsy [4].

Renal artery pseudoaneurysms (RAP) related to biopsy are also rare occurrences, with an incidence of 5% reported in a study looking at 72 percutaneous renal biopsies [5]. This complication results from arterial injury leading to contained hemorrhage within the kidney [5]. Causes of RAPs other than iatrogenic injury from percutaneous renal biopsy include trauma, percutaneous nephrolithotomy, and partial nephrectomy [5]. RAPs are clinically relevant due to their propensity to rupture [6].

In this report, we present a case of multiple pseudoaneurysms in the left kidney as well as a pseudoaneurysm in the upper pole of the right kidney post-percutaneous left kidney biopsy and post-procedure bleeding complication, in a patient with lupus nephritis.

## Case presentation

A 28-year-old African American female patient with a past medical history of systemic lupus erythematosus (SLE) with lupus nephritis class III & V by biopsy in 2017 with progression to chronic kidney disease (CKD) IIIb/IV, as well as SLE-mediated WHO Group 1 pulmonary hypertension requiring a continuous Flolan^TM^ infusion, presented to the hospital for an elective left renal biopsy. Three months prior, the patient had marked proteinuria of 10g/day and was started on voclosporin for lupus nephritis, improving her urinary protein/creatinine ratio to 2 g/day. Two months after starting voclosporin, creatinine increased from 2.6 mg/dl to 4.4 mg/dl. Though voclosporin significantly diminished her estimated daily proteinuria to less than a 1/4 of her recent peak, it also elevated her serum creatinine to 4.4 mg/dL. After the medication was stopped, the creatinine did downtrend to 3.0 mg/dl, then 2.6 mg/dl, but not back to her prior baseline of 1.3 mg/dl. The decision was made to board her for an elective random renal biopsy as there was no longer an explanation for the persistent elevation in creatinine. A month before the biopsy, an ultrasound showed bilateral hyperechoic kidneys, indicative of renal disease and mild bilateral hydronephrosis. On the day of the biopsy, her hemoglobin level was 9.0 g/dl, serum creatinine level was 4.80 mg/dl, blood urea nitrogen (BUN) was 61 mg/dL, and platelet count was 233 x 10^3^/µl.

A preliminary CT of the abdomen demonstrated a targetable left kidney. Under CT/fluoro guidance, a 17 gauge trocar needle was advanced into the left posterior lateral renal cortex of the lower pole. Four 18 gauge core samples were acquired. After the biopsy was complete, Helitene®, a hemostatic agent, was injected as the needle was removed. 

**Figure 1:**
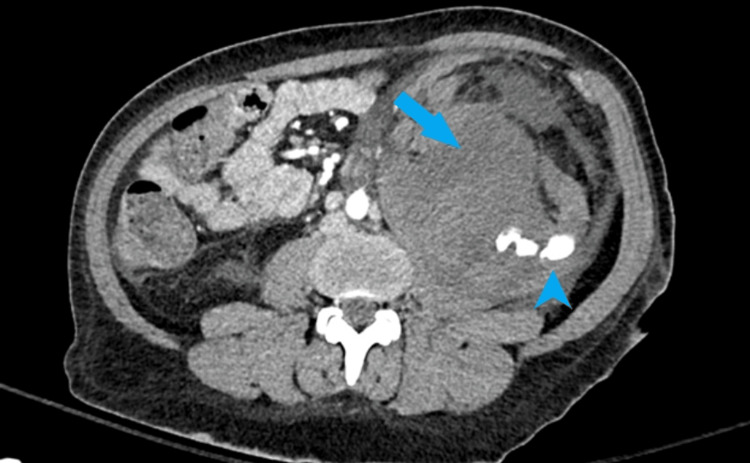
CT scan of the abdomen Active extravasation originating from the left kidney (arrowhead). The arrow shows a complex fluid representing hematoma formation in the kidney and pararenal space (measuring up to 14 cm).

Within the immediate post-procedure period, the patient’s blood pressure dropped from 123/92 to the 80s/50s-90s, and hemoglobin was 5.3 g/dl. An hour after the biopsy, CT at the level of biopsy showed complex fluid collection in the posterior left pararenal space measuring at least 14 cm x 7.7 cm x 12 cm, which extended to the upper pelvis (Figure 1). The fluid collection was causing superior displacement of the left kidney, and there was diminished blood flow to the left kidney due to corresponding subcapsular hematoma and likely intraparenchymal lower pole hematoma. Intravenous (IV) contrast extravasation extended from one of the interlobar lower pole renal arteries into the aforementioned collection. At bedside, in the post-anesthesia care unit, the patient complained of severe abdominal and left back pain consistent with the site of hematoma. 

Emergent angiography of the left renal artery showed brisk active extravasation in one of the branches supplying the inferior lobe of the left kidney. A 2.8 Fr Progreat catheter was used to super-select the interlobar artery, followed by coil embolization utilizing a 2 mm X 4 cm coil, which immediately ceased the observed extravasation. Repeat runs using the microcatheter and the Simmons guiding catheter confirmed successful embolization of the renal artery branch.

Two days after biopsy and embolization, the patient developed 10/10 flank pain, persistent tachycardia (120 beats per minute (bpm)), and required oxygen supplementation. Biopsy results showed global sclerosis of all glomeruli and interstitial fibrosis throughout. On post-operative day 2, laboratory data showed creatinine ranging from 5.6 mg/dl - 4.4 mg/dl, and BUN was 59-56 mg/dL. Spiked fever of 38.6°C and white blood cell count (WBC) trending upwards (18 to 22.7 × 109/L). CT scan without contrast demonstrated loculated hyperdense collection posterior and inferior to the left kidney, measuring 12 cm x 5cm x 21 cm, which was slightly decreased from 12 cm x 7.6 cm x 22 cm since the prior exam. Bilateral delayed nephrograms may be associated with renal failure, nephropathy, or acute tubular necrosis. The patient also developed gross hematuria with the passage of small clots.

**Figure 2:**
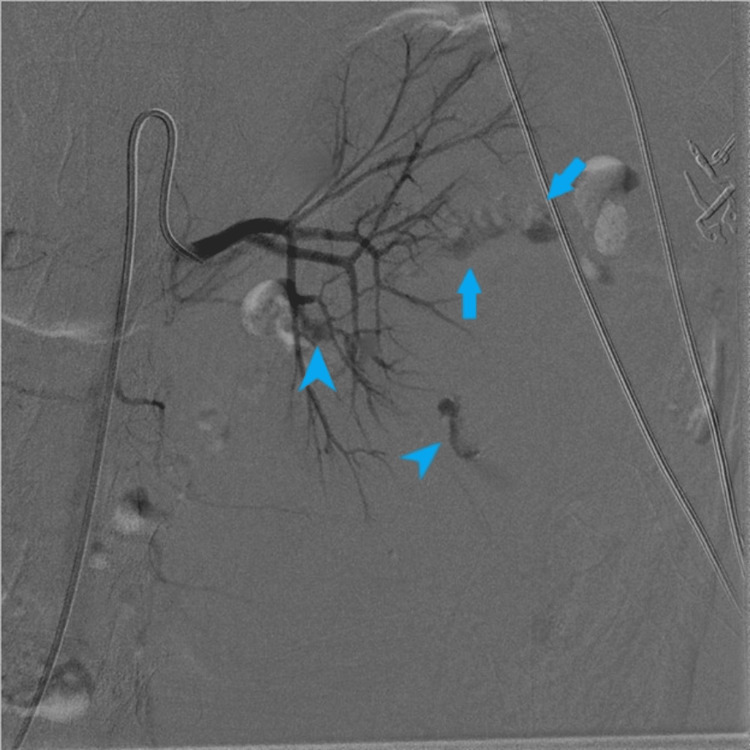
DSA with subtraction imaging of the left kidney Digital subtraction angiography (DSA) with subtraction imaging reveals multiple areas of extravasation in the left kidney, denoted by the arrows. Areas of pseudoaneurysm formation with possible extravasation (arrowheads).

On post-operative day 3, the interventional radiology (IR) service was consulted regarding suspected recurrent renal hemorrhage post-embolization. Angiography showed multiple pseudoaneurysms in the left kidney and a pseudoaneurysm in the right kidney’s upper pole; findings suggesting polyarteritis nodosa (PAN). See Figure 2 for renal angiogram findings. These pseudoaneurysms were not seen on the prior left renal artery angiography on the day of biopsy, likely due to vasoconstriction. Air was also present within the kidney, concerning for infection of hematoma. Initially, it was considered that these aneurysms could be due to an infectious process or underlying lupus. No intervention was performed since there was no identifiable source for the embolization on the second arteriogram.

## Discussion

The risk of bleeding complications and renal pseudoaneurysms in patients with SLE is not well defined in the literature [7]. The most severe complications from pseudoaneurysms require blood transfusion and arterial embolization under fluoroscopy or even nephrectomy [7]. This case report presents a patient with lupus nephritis who presented to the hospital for a left kidney renal biopsy. Multiple pseudoaneurysms/microaneurysms were found in both kidneys, in the upper pole on the left, away from the biopsy site. A pseudoaneurysm was also found in the kidney, in which biopsy was not performed. These findings may suggest polyarteritis nodosa (PAN) or true renal lupus vasculitis (TRLV). True renal vasculitis is a rare lupus vascular lesion that involves small arteries and, most frequently, the intralobular arteries [8]. TRLV and PAN cannot be distinguished histologically [8]. The presence of vasculitis in a lupus nephritis biopsy is associated with a worse prognosis and the risk of end-stage renal disease [8]. On the other hand, PAN is a systemic necrotizing vasculitis of small or medium-sized arteries [8]. PAN findings include microaneurysms stemming from weakened arterial wall points [8]. 

Renal pseudoaneurysms are rare vascular lesions that can lead to life-threatening hemorrhage, and they must be differentiated from a true aneurysm [9]. An aneurysm is a circumscribed dilation of an artery surrounded by intima, media, and adventitia, whereas a pseudoaneurysm forms as a consequence of an injury to one or more layers of the arterial wall [5]. A pseudoaneurysm contains a hematoma surrounded by adventitia and perivascular tissue communicating with an artery or vein [9]. Compressive forces slow down the bleeding at the site of injury or disease, and then reactive fibrosis encapsulates the hemorrhage forming the aneurysmal sac [5,9]. Clinically, pain and hematuria are common symptoms [5]. Patients are usually unstable, tachycardic, anemic, and hypotensive [5]. However, they can vary in presentation, ranging from asymptomatic, perinephric hematoma, abdominal mass, flank pain, and hypovolemic shock. There should be a strong suspicion of a pseudoaneurysm when a patient develops flank pain, hypotension, and hematuria following intervention [9]. These symptoms were found in the case of our patient in this report [9]. In addition, pseudoaneurysms have a saccular shape and usually involve the extraparenchymal and intraparenchymal renal arteries and their branches [9]. Presentation varies and can be an isolated finding, cluster, or multiple throughout the parenchyma [9]. 

Multiple intraparenchymal pseudoaneurysms mainly stem from inflammatory changes of the vessel wall that progress to form microaneurysms [9]. Though pseudoaneurysms are usually caused by penetrating trauma, due to the patient’s lupus nephritis, lupus vasculitis is the most likely cause of her bilateral pseudoaneurysms. Vasculitides’ inflammatory processes lead to necrosis and destruction of the vessel wall, leading to pseudoaneurysm formation [9]. Symptoms may occur days to years later when a renal vascular procedure is the underlying cause [5]. Nevertheless, the clinical signs are unreliable and do not mirror the severity and etiology of the lesion [5]. 

The literature is limited regarding the diagnosis of renal pseudoaneurysms [5]. There need to be more cases to develop official guidelines [5]. Currently, enhanced CT is the imaging of choice [5,10]. In patients with impaired renal function, MR angiography without contrast or with non-gadolinium contrast, such as ferumoxytol, may be used [10]. Cura et al. state that contrast-enhanced computed tomography angiography (CTA) provides better anatomical detail of the renal arteries compared to sonography [9]. 

Treatment depends on etiology, location, size, and symptoms, and it is indicated in ruptured aneurysms and actively bleeding pseudoaneurysms [5,9]. Angioembolization is the preferred treatment, given this procedure's low risk of long-term renal impairment [5]. Moreover, pseudoaneurysms larger than 2 cm present a high risk for rupture, and treatment is recommended with transcatheter embolization, whereas smaller pseudoaneurysms may resolve spontaneously [10]. If an interventional radiologist is not available, surgery must be considered. However, this approach must be considered after failing embolization [5]. Nephrectomy is considered next after every other intervention has failed [5]. 

In addition, patients with SLE often have coexistent hematologic abnormalities such as anemia, thrombocytopenia, and antiphospholipid syndrome [7]. Hence, these patients may be at an increased risk for post-biopsy complications. Chen et al. documented that significant complications in patients with SLE are relatively low, at 2.7% [7]. Patients with platelet counts <150,000 cells/mm^3^ were 30 times more likely to develop biopsy-related major complications and four times more likely to have any biopsy-related bleeding complications [7]. Female gender, younger age, and a higher partial thromboplastin time were associated with increased risk for bleeding [7]. An elevated serum creatinine, high systolic blood pressure, or use of a larger needle gauge are also associated with an increased risk of hemorrhage [3]. The patient in this case report did have a high creatinine level; however, her platelet count was within normal limits. In general, despite renal biopsies being relatively safe procedures, hematuria and hematomas cannot be completely avoided [11]. Superselective renal embolization is the best approach for renal injury; hence, this was used in this patient when a hematoma occurred [11]. Abnormal vessels are located, and occlusion is achieved without damaging normal renal parenchyma [11]. 

Recommendations to decrease bleeding and pseudoaneurysm complications in patients with SLE may include a platelet count of 100,000 cells/mm^3^ before renal biopsy [7]. In addition, pseudoaneurysm should always be considered in the differential diagnosis in patients who develop hemorrhage associated with interventions such as percutaneous kidney biopsy, especially in patients with SLE [5]. 

## Conclusions

In conclusion, we showed a case of a 28-year-old woman with lupus nephritis who presented for a percutaneous renal biopsy. Follow-up imaging showed bleeding and left perinephric hematoma post-biopsy, with the incidental finding of pseudoaneurysms in both kidneys. Hence, renal pseudoaneurysm should be high in the differential for a patient with post-percutaneous biopsy who presents with anemia, flank pain, or hematuria.
